# Increase in sickness absence with psychiatric diagnosis in Norway: a general population-based epidemiologic study of age, gender and regional distribution

**DOI:** 10.1186/1741-7015-4-19

**Published:** 2006-08-22

**Authors:** Gunnel Hensing, Lena Andersson, Sören Brage

**Affiliations:** 1 Department of Public Health and Community Medicine, Institute of Medicine, The Sahlgrenska Academy at Göteborg University, Göteborg, Sweden; 2 Department of General Practice and Community Medicine, University of Oslo, Oslo, Norway

## Abstract

**Background:**

The aim of this study was to assess the incidence of sickness absence with psychiatric diagnoses from 1994–2000, and the distribution across gender, age groups, diagnostic groups and regions in a general population.

**Methods:**

The population at risk was defined as all individuals aged 16–66 years who were entitled to sickness benefits in 1994, 1996, 1998 and 2000 (n = 2,282,761 in 2000). All individuals with a full-time disability pension were excluded. The study included approximately 77% of the Norwegian population aged 16–66 years. For each year, the study base started on 1 January and ended on 31 December. Individuals that were sick-listed for more than 14/16 consecutive days with a psychiatric diagnosis on their medical certificate were selected as cases. Included in this study were data for Norway, the capital city Oslo and five regions in the southeast of the country.

**Results:**

Sickness absence with psychiatric diagnoses increased in all age groups, in women and men, and in all regions. At the national level, the cumulative incidence increased in women from 1.7% in 1994 to 4.6% in 2000, and in men from 0.8% in 1994 to 2.2% in 2000. The highest cumulative incidence was found in middle-aged women and men (30–59 years). Women had a higher incidence than men in all stratification groups. The cumulative incidences in 2000 varied between 4.6% to 5.6% in women in the different regions, and for men the corresponding figures were 2.1% to 3.2%. Throughout the four years studied, women in Oslo had more than twice as high incidence levels of sickness absence with alcohol and drug diagnoses as the country as a whole. There were some differences between regions in sickness absence with specific psychiatric diagnoses, but they were small and most comparisons were non-significant.

**Conclusion:**

Sickness absence with psychiatric diagnoses increased between 1994 and 2000 in Norway. The increase was highest in the middle-aged, and in women. Few regional differences were found. That the increase pervaded all stratification groups supports general explanations of the increase, such as changes in attitudes to psychiatric disorders in both patients and doctors, and increased mental distress probably associated with societal changes at a more structural level.

## Background

Psychiatric disorders have always been one of the most common diagnostic groups in sickness absence, and in recent years there has been an increase in cases [1-4]. Whether this represents a true increase in the prevalence of psychiatric disorders or an adjustment in sickness absence to the real occurrence of lowered work capacity due to psychiatric disorders has been the subject of discussion [5,6]. General population-based assessments of cumulative incidence of sickness absence with psychiatric disorders have varied from 2.1% for women and 1.3% for men in Sweden in 1985 to 3.53% and 1.66% in Norway in 1998 [7-9]. A higher incidence among women has been reported by several studies [7-10]. Contrary to most other diagnostic groups, the mean number of sick leave days with psychiatric disorders is similar among women and men.

In the Whitehall II study on civil servants aged 35–55 years Stansfeld et al[6] found a social gradient in sickness absence with psychiatric disorders and a higher incidence in widowers, single men and divorced women. In occupational groups that were extremely male or female dominated, the sex in the minority had a higher incidence of sick leave with psychiatric disorders [7]. Stansfeld et al. [6] found decreased sickness absence with psychiatric disorders amongst men with high decision latitude at work, varied and challenging work, and good social support from colleagues, workmates and managers. Varied and challenging work, good social support and information from managers predicted low sickness absence with psychiatric disorders among women. Finally, high demands at work were associated with increased sickness absence with psychiatric disorders among men. Stansfeld et al. [11] also studied factors outside work and found that negative social support was associated with an increased risk for long sickness absence and that material problems were associated with short sick-leave spells.

The economic consequences of sickness absence with psychiatric disorders are important. The total National Insurance expenditure for psychiatric disorders in sickness absence, medical rehabilitation and disability pension in Norway adds up to EUR 2,676 mill. in 2004 which corresponds to 27% [12]. Few specific studies on sickness absence with psychiatric disorders have been performed with the general population as a study base, and thus little is known about its occurrence and distribution [13]. Several of the published studies in this area analyse data collected in the middle of the 1980s, which implies a need for more recent information. The possibility of carrying out such studies is particularly good in Norway where a nationwide sickness absence register has been developed. The aim of this study was thus to assess the incidence of sickness absence with psychiatric diagnoses from 1994–2000, and the distribution across gender, age groups, diagnostic groups and regions in a general population. The analysis of differences between these strata can contribute to explaining the mechanisms involved in the increase of sickness absence with psychiatric diagnoses.

## Methods

This study is part of a project with the overall aim of describing and explaining regional differences in sickness absence and disability pension with psychiatric diagnoses in coastal and rural regions of southwest Sweden and southern Norway [14,15]. These regions surround the Skagerrak, which was a connecting link area during the period when fishing and shipping were important industries, up until the end of the 1970s. Thus, there is a common historical and cultural tradition, including a rigorous and conservative Protestantism. Intensive trade over the national boarders still exists, both in goods and in the labour force. Family ties across borders have been common.

### The sickness benefit scheme

Information, including diagnosis, on all benefits from the Sickness Benefit Scheme is registered by the Norwegian National Insurance Administration (NIA) [16]. All employed, self-employed and temporarily unemployed individuals in Norway with an annual income over EUR 2,964 are compulsory members of the Sickness Benefit Scheme. Many students thus are excluded from the scheme. Part-time workers and persons working parts of the year are included. During the study period 1994–2000, civil servants (approximately 8% of the work force) were not part of the Sickness Benefit Scheme, as they received their benefits directly from their employer [9]. In Norway, 100% of the sick-listed person's normal income is compensated for, up to a maximum of approximately EUR 37,000. Benefits are paid from the first day of absence, but the initial 14 days (16 days after 1 April 1998) are paid by the employer. Benefits are paid by the NIA from day 15/17. From day 4 of an absence period, a doctor's certificate is required. This certificate can be provided by any doctor, but is most frequently given by general practitioners. The doctor must provide both a diagnosis and a diagnostic code according to ICD or ICPC on the certificate. Only the main diagnostic code is entered into the register. The maximum duration of sickness benefit is one year. After that, rehabilitation allowances or disability pensions are given.

### Study base

The population at risk was defined as all individuals aged 16–66 years who were entitled to sickness benefits in 1994, 1996, 1998 and 2000, also including part-time and seasonal workers. All individuals with a full-time disability pension were excluded. The study includes approximately 77% of the Norwegian population aged 16–66 years. For each year, the study base started on 1 January and ended on 31 December. Individuals who were sick-listed at least once for more than 14/16 consecutive calendar days with a psychiatric diagnosis on their sickness certificate were selected as cases. The diagnoses were grouped into seven subgroups (Table 1).

Included in this study were data for Norway, the capital city Oslo and five regions in the southeast of the country close to coastal regions with connections to the west coast of Sweden. High levels of psychiatric morbidity, monitored by different health indicators such as sickness absence, disability pension and prescription of psychopharmacological drugs, have been found in these regions on both sides of the border [17].

### Sickness absence measures and statistics

Sickness absence can be measured in several ways, and comparisons between studies have been difficult as researchers use different measures that are often not well defined [18]. In this study we analysed cumulative incidence, which we defined as:

No. of individuals with at least one sickness absence episode initiated in each year studiedNo. of individuals entitled to sickness benefits during that year
 MathType@MTEF@5@5@+=feaafiart1ev1aaatCvAUfKttLearuWrP9MDH5MBPbIqV92AaeXatLxBI9gBamXvP5wqSXMqHnxAJn0BKvguHDwzZbqegyvzYrwyUfgarqqtubsr4rNCHbGeaGqipGI8VfYxH8qipiYdHaVhbbf9v8qqaqFr0xc9pk0xbba9q8WqFfeaY=biLkVcLq=JHqVepeea0=as0db9vqpepesP0xe9Fve9Fve9GapdbaqaaeGacaGaaiaabeqaamqadiabaaGcbaWaaSaaaeaacqqGobGtcqqGVbWBcqqGUaGlcqqGGaaicqqGVbWBcqqGMbGzcqqGGaaicqqGPbqAcqqGUbGBcqqGKbazcqqGPbqAcqqG2bGDcqqGPbqAcqqGKbazcqqG1bqDcqqGHbqycqqGSbaBcqqGZbWCcqqGGaaicqqG3bWDcqqGPbqAcqqG0baDcqqGObaAcqqGGaaicqqGHbqycqqG0baDcqqGGaaicqqGSbaBcqqGLbqzcqqGHbqycqqGZbWCcqqG0baDcqqGGaaicqqGVbWBcqqGUbGBcqqGLbqzcqqGGaaicqqGZbWCcqqGPbqAcqqGJbWycqqGRbWAcqqGUbGBcqqGLbqzcqqGZbWCcqqGZbWCcqqGGaaicqqGHbqycqqGIbGycqqGZbWCcqqGLbqzcqqGUbGBcqqGJbWycqqGLbqzcqqGGaaicqqGLbqzcqqGWbaCcqqGPbqAcqqGZbWCcqqGVbWBcqqGKbazcqqGLbqzcqqGGaaicqqGPbqAcqqGUbGBcqqGPbqAcqqG0baDcqqGPbqAcqqGHbqycqqGSbaBcqqGLbqzcqqGKbazcqqGGaaicqqGPbqAcqqGUbGBcqqGGaaicqqGLbqzcqqGHbqycqqGJbWycqqGObaAcqqGGaaicqqG5bqEcqqGLbqzcqqGHbqycqqGYbGCcqqGGaaicqqGZbWCcqqG0baDcqqG1bqDcqqGKbazcqqGPbqAcqqGLbqzcqqGZbWCaeaacqqGobGtcqqGVbWBcqqGUaGlcqqGGaaicqqGVbWBcqqGMbGzcqqGGaaicqqGPbqAcqqGUbGBcqqGKbazcqqGPbqAcqqG2bGDcqqGPbqAcqqGKbazcqqG1bqDcqqGHbqycqqGSbaBcqqGZbWCcqqGGaaicqqGLbqzcqqGUbGBcqqG0baDcqqGPbqAcqqG0baDcqqGSbaBcqqGLbqzcqqGKbazcqqGGaaicqqG0baDcqqGVbWBcqqGGaaicqqGZbWCcqqGPbqAcqqGJbWycqqGRbWAcqqGUbGBcqqGLbqzcqqGZbWCcqqGZbWCcqqGGaaicqqGIbGycqqGLbqzcqqGUbGBcqqGLbqzcqqGMbGzcqqGPbqAcqqG0baDcqqGZbWCcqqGGaaicqqGKbazcqqG1bqDcqqGYbGCcqqGPbqAcqqGUbGBcqqGNbWzcqqGGaaicqqG0baDcqqGObaAcqqGHbqycqqG0baDcqqGGaaicqqG5bqEcqqGLbqzcqqGHbqycqqGYbGCaaaaaa@FE9B@

Cumulative incidence was expressed as a percentage (%). Comparisons of cumulative incidences were presented as ratios with 95% confidence intervals (hereafter 95%CI). Norway was used as reference for the analysis of the regional/national ratio. The ratio was calculated by dividing the cumulative incidence of sickness absence for a region with the cumulative incidence of Norway as a whole. All data presented were age-adjusted and the whole population entitled to benefits was used as standard. All analyses were performed with SPSS and Excel software programs.

## Results

In the years examined, we found an increase in sickness absence with psychiatric diagnoses across all age groups. At the national level the cumulative incidence increased from 1.7% in 1994 to 4.6% in 2000 in women, and for men the corresponding figures were 0.8% and 2.2%. In 1994, the cumulative incidence in women aged 16–29 years was 0.8% (95% CI) and this had increased to 2.6% six years later (Table 2). The highest cumulative incidence was found in women aged 40–49 years. Of women in this age group in Oslo, 6.5% were sick-listed at least once with a sick-leave spell over 16 days with a psychiatric diagnosis in 2000. Women aged 30–39 years and 50–59 years also had high incidences. Among men the cumulative incidence increased in all age groups (Table 3). As for women, men in the age groups from 30 to 59 years had the highest cumulative incidence. Throughout, the levels of cumulative incidences in men were lower than for women. Furthermore, the increase of sickness absence was greater for women than for men in all age groups and in all regions studied. The lowest increase was for the youngest and oldest age groups for both men and women.

The cumulative incidence of sickness absence with psychiatric diagnoses increased in all regions included in the study (Table 4). In 1994, the regional differences were large, with a higher cumulative incidence for women as well as for men in Oslo (Table 3) and the two rural counties of Aust-Agder and Vest-Agder (Table 4). In 2000, there were some changes in regional differences (Tables 2 and 4): women in Telemark had the highest cumulative incidence, but Oslo, Aust-Agder and Vest-Agder also had high proportions of people on sick leave, with figures of around 5% of the female population sick-listed at least once. For men, Vest-Agder had the highest cumulative incidence, but as for women the regional differences were smaller in the year 2000. The highest increase in sickness absence was found in women in Telemark, with an increase from a cumulative incidence of 1.4% in 1994 to 5.6% in 2000.

The highest cumulative incidence of sickness absence in different psychiatric diagnostic groups was found in depression in both women and men (Table 5) with an increase from 1.21% to 3.01% in women. Figures for most other diagnostic groups were low. For example, the cumulative incidence in sickness absence with alcohol and drug disorders varied from 0.06% to 0.09% in men.

## Discussion

### Changes in attitudes to psychiatric disorders and increased distress?

One finding of this study was that the increase in sickness absence with psychiatric diagnoses occurred in both men and women, in all age groups, in all regions and in the diagnostic groups with the most sick-listed. This pervading increase implies that underlying causes were related to factors that affect men and women, different age groups and different regions in a similar way. Stansfeld et al. [6] suggested different explanations for the increase of sickness absence with psychiatric diagnoses. Among these were changes in attitudes to psychiatric disorders in the general population, affecting both patients and doctors. With a less stigmatising attitude to psychiatric disorders, more patients disclose such symptoms, doctors become more inclined to identify these diagnoses, and both patients and doctors accept to a greater extent the recording of a psychiatric diagnosis on the certificate. Stansfeld et al. [6] further suggested that the availability to physicians of better drugs (e.g. SSRI drugs) for the treatment of psychiatric disorders might lead to improved and increased identification of psychiatric disorders. Furthermore, there has been a steady increase in the proportion of sickness absence spells with a psychiatric diagnosis. In 1995, 11.6% of sickness episodes longer than 16 days had a psychiatric diagnosis; in 2000, 15.7%. In the same period the total number of sickness absence spells increased from 316,114 to 501,708. Thus, both overall and psychiatric sickness absence increased in the study period, but psychiatric sickness absence had a greater increase [19].

The increase in sickness absence with psychiatric disorders might also reflect an increase of psychiatric disorders or mental distress. Many researchers and public health reports in Scandinavia have expressed doubt about a significant increase in psychiatric disorders in the population, but have confirmed increased distress and a decrease in psychological wellbeing in general [20]. Increased stress and decreased psychological wellbeing might lead to a decline in mental as well as physical health, such as increased pain, headache, and other musculoskeletal and gastrointestinal symptoms. As mentioned in the introduction, Sandanger et al. [5] found that the relation between health problems and sickness absence differed between psychiatric disorders and musculoskeletal disorders, with a suggested underreporting of sickness absence with psychiatric diagnoses. We therefore think that the most plausible explanations for the pervading increase in sickness absence with psychiatric disorders are an increase in stress in the population, and changes in attitudes of both patients and physicians leading to a higher number of people being sick-listed with a psychiatric diagnosis than previously. Whether this merely reflects a change in certification behaviour or whether there has also been a change in how individuals express their stress and low psychological wellbeing via different symptoms is difficult to ascertain.

### A higher incidence among women remains and differences between the sexes increase

Another important finding of this study was that the cumulative incidence as well as the increase in cumulative incidences of sickness absence was highest in women aged 30–59 years. In the systematic review of research on sickness absence with psychiatric diagnoses, the higher incidence of women was the only finding that could be identified as evidence-based [13]. The higher incidence corresponds well to the higher prevalence of anxiety and depressive disorders found in women compared to men [21]. Several other explanations have been put forward, such as gender differences in health-seeking behaviour, in symptom presentation, symptom interpretation of both patient and physician, in psychological development and socialisation, and in exposure to risk factors such as sexual and violent abuse, other traumatic events, sole responsibility for children, and poverty [21-24]. The finding that women not only had a higher cumulative incidence but also the largest increase is important from a public health point of view, as it means that an increasing proportion of Norwegian women are affected by absence from work relating to mental illness. It is not likely that the change in attitudes discussed above had a specific effect on women aged 30–59, so we must look for other explanations. In both men and women the increase was greatest among ages when activity in the labour market is highest in combination with responsibilities for family and household, with increased stress in both sexes [25]. However, the effect of the combination of paid and unpaid work seems to be higher in women, even if several studies have also shown that the health effect of combining paid and unpaid work is beneficial for women's (and men's) health. During the 1990s, workplaces in Norway, as in the rest of Europe, increased their efficiency by downsizing organisations, and this also affected typically female-dominated workplaces in health, child and elderly care, service professions and education, with increased demands placed both on employees and managers in these organisations. Organisational changes, management quality, low control and low social support are factors that have been shown to be associated with increased sickness absence in women [1,6,26,27]. It is very plausible that the high increase of sickness absence with psychiatric diagnoses in women from 1994 to 2000 reflects increased demands in female-dominated workplaces, in combination with an unchanged distribution of unpaid work in the family and household [25]. The increase in men probably reflects the same kind of increased demands but without the specific problems associated with the public sector (low wages, low control, less skilled managers, high emotional stress related to work with people rather than things) and without the high unpaid workload still associated with the female role in society. However, it is important to remember that we have no data on occupations in this study, and some of the gender differences might be explained by different distribution over socio-economic strata in men's and women's occupations. The role of work in sickness absence with psychiatric disorders need more attention in future studies [1], and of specific interest is health-related selection that can contribute to bias in cross-sectional studies on gender differences.

### An inclusive labour market

Another possible explanation for the increasing rates is that during the study period Norway had falling unemployment rates, from 4.9 % in 1995 to 3.4 % in 2000 [28]. With lower unemployment rates the possibility for individuals with health problems to get a job is higher. The proportion of individuals with health problems in the work force thus increase which might contribute to high sickness absence rates. Individuals with higher vulnerability due to health problems are likely to be the most sensitive to a more demanding labour market. It is possible that individuals with a vulnerability for mental illness are more sensitive to such changes, especially in a work life, which places increasing demands on the individuals to perform well both regarding social and cognitive functions [13].

### Regional inequity, selection or differences in health?

The capital Oslo had high cumulative incidences. An increased risk for alcohol and drug problems in urban areas as well as an increased risk for psychoses might contribute to these findings, even if these diagnostic groups had low cumulative incidences. Stansfeld et al. [6] found an underreporting of psychoses on medical certificates so people with alcohol and drug problems and psychoses might be sick-listed with other diagnoses. The higher urban incidence has been explained by selection of individuals with these health problems into large cities, or by exposure to living conditions more characterised by poverty, low social support, high criminality and access to drugs [29-31]. Andersson et al. [15] found an increased risk for disability pensions with psychiatric diagnoses in men in Oslo, but not in women. Psychoses and substance abuse among men was in that study found to be more prevalent in Oslo, while the distribution of neurotic and somatoform disorders showed no regional differences. For women there were no regional differences in psychoses and alcohol and drug disorders, while neurotic disorders were more prevalent in semi-rural areas. It is possible that the high sickness absence found among women in Oslo will lead to increased disability pensions, as several studies have found that an important risk factor for future disability pension is earlier sickness absence [32,33]. To explain regional differences, information on the distribution over occupations in different regions would have been helpful in order to add in controls for possible selection or confusion associated with labour market factors and occupational roles. Another factor of interest is the access to mental health care and rehabilitation.

### Methodological considerations

This study was performed based on a national social insurance register covering almost the whole Norwegian population. Apart from earlier studies in Norway by Hensing et al. [13] and Nystuen et al. [9], there are no other general population-based studies on sickness absence with psychiatric diagnoses that have included such a large number of individuals. As the major part of the population is included, the selection bias common in studies based on specific occupations or workplaces is not an issue in this study [13]. The validity of diagnoses on sickness certificates has not been studied to a very large extent, but it can be hypothesised that the specificity of psychiatric diagnoses (considered as a diagnostic group) is high. Brage et al. [34] found few changes of psychiatric diagnoses in the long-term sick-listed. It was more common that individuals originally sick-listed with musculoskeletal diagnoses were later sick-listed with psychiatric diagnoses. The validity between different diagnoses is probably lower[13]. Stansfeld et al. [6,11], as mentioned, found an underreporting of psychoses on sickness certificates, and Hensing et al. [35] did not find any alcohol diagnoses on certificates in a study of sickness absence in women with alcohol problems.

The limitation of this register-based study is that we have access to a limited number of factors to study. We have no information on occupations, working conditions or family situation, which could have contributed to explaining the differences found between women and men more fully.

In the study period 1994–2000, sickness absence episodes shorter than 14 (16) days were not recorded in the registers, and could thus not be included in the study. The duration of sickness absence episodes varies with diagnosis, and is longer for psychiatric diagnosis than other diagnostic groups (with the exception of cardiovascular disorders), indicating that shorter sickness absence episodes are infrequent in persons with psychiatric diagnoses [12]. The exclusion of episodes shorter than 14 days therefore gives an underestimate of the real cumulative incidence of sickness absence, but is of limited importance.

## Conclusion

Sickness absence with psychiatric diagnoses more than doubled between 1994 and 2000 in Norway. This considerable increase was highest in the middle aged, and in women. Few regional differences were found. That the increase pervaded all stratification groups supports general explanations for the increase such as changes in attitudes to psychiatric disorders in both patients and physicians, and an increased mental distress possibly associated with societal changes at a more structural level. Future studies should address the persisting differences between women and men in sickness absence with psychiatric diagnoses.

## Competing interests

The author(s) declare that they have no competing interests.

## Authors' contributions

The work with this study has been a true collaboration and GH, LA, SB have all contributed to the design, the analyses, the interpretation of data and the writing of the manuscript.

## Pre-publication history

The pre-publication history for this paper can be accessed here:



## Figures and Tables

**Table 1:** Sickness absence diagnoses according to the International Classification of Primary Care (ICPC), Norway

Diagnostic group	Diagnosis code (ICPC-1)^a ^1994 and 1996	Diagnosis code (ICPC-2)^a ^1998 and 2000	Corresponding codes in ICD-10
Psychoses	P72, P73, P98	P72, P73, P98	F20–F31, F35–F39
Anxiety	P01, P74	P01, P74	F40–F43
Neurotic conditions	P02, P04, P06–P10, P20, P25, P27–P29, P75, P78, P79, P99	P02, P04, P06–P10, P20, P25, P27–P29, P75, P78, P79, P82, P99	F44–48, F99
Depression	P03, P76, P77	P03, P76, P77	F32–F34
Personality disorders	P80	P80	F60–F69
Alcohol/drug abuse	P15- P19	P15- P19	F10–F19
Excluded*	P05, P11–P13, P21 -P24, P70, P71, P85	P05, P11–P13, P21–P24, P70, P71, P81, P85	F00–F09, F70–F98

* Diagnostic groups excluded were dementia, organic psychoses, mental retardation and child and adolescent psychiatry.

**Table 2:** Cumulative incidence of sickness absence with psychiatric disorders in different age groups in Norway and Oslo, 1994, 1996, 1998 and 2000, women.

		Norway			Oslo			
Age group	Year	No. of sickness insured women	Cumulative incidence (95% CI)	Change 1994–2000 +/-	No. of sickness insured women	Cumulative incidence (95% CI)	Change 1994 – 2000 +/-	Regional/national ratio (95% CI)

16–29	1994	258,213	0.8 (0.8–0.8)		28,033	1.2 (1.2–1.2)		1.48 (1.32–1.66)
	1996	261,990	1.2 (1.2–1.2)		29,218	1.8 (1.8–1.8)		1.52 (1.39–1.67)
	1998	273,748	1.9 (1.9–1.9)		31,738	2.4 (2.4–2.4)		1.26 (1.17–1.36)
	2000	277,707	2.6 (2.6–2.6)	+1.7	33,574	3.1 (3.1–3.1)	+1.9	1.22 (1.14–1.30)

30–39	1994	236,471	2.1 (2.1–2.1)		27,007	2.5 (2.5–2.5)		1.23 (1.14–1.33)
	1996	247,213	2.9 (2.9–2.9)		30,077	3.4 (3.4–3.4)		1.16 (1.08–1.24)
	1998	258,184	4.1 (4.1–4.1)		31,539	4.4 (4.4–4.4)		1.07 (1.01–1.13)
	2000	272,355	5.3 (5.3–5.3)	+3.2	32,877	5.4 (5.4–5.4)	+2.8	1.02 (1.97–1.07)

40–49	1994	231,885	2.3 (2.3–2.3)		20,659	3.0 (3.0–3.0)		1.29 (1.19–1.40)
	1996	237,976	3.3 (3.3–3.3)		21,733	4.1 (4.1–4.1)		1.26 (1.18–1.35)
	1998	242,663	4.8 (4.8–4.8)		22,427	5.3 (5.3–5.3)		1.09 (1.03–1.16)
	2000	252,498	5.9 (5.9–5.9)	+3.6	24,337	6.5 (6.5–6.5)	+3.5	1.10 (1.04–1.15)

50–59	1994	142,788	2.0 (2.0–2.0)		13,408	2.5 (2.5–2.5)		1.22 (1.09–1.37)
	1996	165,560	2.9 (2.9–2.9)		15,923	3.5 (3.5–3.5)		1.18 (1.08–1.29)
	1998	184,511	4.1 (4.1–4.1)		17,629	4.3 (4.3–4.3)		1.05 (0.98–1.13)
	2000	202,207	5.1 (5.1–5.1)	+3.1	19,815	5.5 (5.5–5.5)	+3.1	1.09 (1.02–1.16)

60–66	1994	53,327	1.9 (1.9–1.9)		5,783	2.4 (2.4–2.4)		1.16 (0.99–1.36)
	1996	54,043	2.6 (2.6–2.6)		5,825	3.0 (3.0–3.0)		1.03 (0.89–1.20)
	1998	55,522	3.3 (3.3–3.3)		5,725	3.4 (3.4–3.4)		1.06 (0.93–1.21)
	2000	58,656	3.7 (3.7–3.7)	+1.8	6,210	4.0 (4.0–4.0)	+1.5	1.38 (1.17–1.64)

Total	1994	922,684	1.8 (1.7–1.8)		94,890	2.3 (2.2–2.4)		1.29 (1.22–1.34)
	1996	966,782	2.5 (2.5–2.5)		102,776	3.1 (3.0–3.2)		1.24 (1.20–1.29)
	1998	1,014,628	3.6 (3.6–3.7)		109,058	4.0 (3.9–4.1)		1.10 (1.06–1.13)
	2000	1,063,423	4.6 (4.6–4.7)	+2.8	116,813	5.0 (4.9–5.1)	+2.7	1.09 (106.–1.12)

**Table 3:** Cumulative incidence of sickness absence with psychiatric disorders in different age groups in Norway and Oslo, 1994, 1996, 1998 and 2000, men.

		Norway			Oslo			
Age group	Year	No. of sickness insured men	Cumulative incidence (95% CI)	Change 1994 – 2000 +/-	No. of sickness insured men	Cumulative incidence (95% CI)	Change 1994 – 2000 +/-	Region/nation ratio (95% CI)

16–29	1994	295,134	0.4 (0.4–0.4)		25,452	0.6 (0.6–0.6)		1.38 (1.17–1.64)
	1996	299,080	0.6 (0.6–0.6)		27,663	0.8 (0.8–0.8)		1.32 (1.15–1.51)
	1998	308,784	1.1 (1.1–1.1)		31,619	1.3 (1.3–1.3)		1.23 (1.11–1.36)
	2000	306,750	1.5 (1.5–1.5)	+1.1	35,077	1.6 (1.6–1.6)	+1.0	1.07 (0.98–1.17)

30–39	1994	282,087	0.9 (0.9–0.9)		32,005	1.2 (1.2–1.2)		1.29 (1.16–1.43)
	1996	293,613	1.3(1.3–1.3)		35,810	1.6 (1.6–1.6)		1.23 (1.13–1.35)
	1998	304,909	2.0 (2.0–2.0)		38,704	2.0 (2.0–2.0)		1.02 (0.94–1.10)
	2000	316,319	2.6 (2.6–2.6)	+1.7	42,992	2.4 (2.4–2.4)	+1.2	0.93 (0.87–0.99)

40–49	1994	269,518	1.1 (1.1–1.1)		23,629	1.6 (1.6–1.6)		1.50 (1.34–1.67)
	1996	273,295	1.5 (1.5–1.5)		25,048	2.1 (2.1–2.1)		1.46 (1.34–1.60)
	1998	275,377	2.1 (2.1–2.1)		25,924	2.7 (2.7–2.7)		1.31 (1.22–1.42)
	2000	282,540	2.7 (2.7–2.7)	+1.6	28,734	3.1 (3.1–3.1)	+1.5	1.16 (1.08–1.24)

50–59	1994	171,782	0.9 (0.9–0.9)		14,539	1.3 (1.3–1.3)		1.39 (1.20–1.62)
	1996	198,071	1.3 (1.3–1.3)		17,909	1.7 (1.7–1.7)		1.30 (1.15–1.46)
	1998	220,152	1.8 (1.8–1.8)		20,287	2.0 (2.0–2.0)		1.12 (1.01–1.24)
	2000	239,252	2.3 (2.3–2.3)	+1.4	22,901	2.4 (2.4–2.4)	+1.1	1.04 (0.96–1.14)

60–66	1994	71,470	0.9 (0.9–0.9)		6,462	1.1 (1.1–1.1)		1.26 (0.99–1.62)
	1996	70,288	1.2 (1.2–1.2)		6,350	1.5 (1.6–1.6)		1.34 (1.08–1.65)
	1998	71,952	1.6 (1.6–1.6)		6,311	1.9 (1.9–1.9)		1.21 (1.01–1.46)
	2000	74,477	1.9 (1.9–1.9)	+1.0	7,029	2.1 (2.1–2.1)	+1.0	1.14 (0.96–1.35)

Total	1994	1,089,991	0.8 (0.8–0.8)		102,087	1.1 (1.0–1.2)		1.39 (1.30–1.47)
	1996	1,134,437	1.1 (1.1–1.2)		112,780	1.5 (1.4–1.6)		1.34 (1.27–1.41)
	1998	1,181,174	1.7 (1.7–1.7)		122,845	2.0 (1.9–2.1)		1.68 (1.12–1.22)
	2000	1,219,338	2.2 (2.2–2.3)	+1.4	136,733	2.4 (2.3–2.4)	+1.3	1.05 (1.01–1.09)

**Table 4:** Cumulative incidence and regional/national ratio of sickness absence with psychiatric disorders in 1994, 1996, 1998 and 2000 (age-adjusted data).

Region	Year	No. of sickness insured women	Cumulative incidence (%) (95% CI)	Change 1994 – 2000 +/-	Regional/national ratio* (95% CI)	No. of sickness insured men	Cumulative incidence (%) (95% CI)	Change 1994 – 2000 +/-	Regional/national ratio* (95% CI)
Östfold	1994	48,864	1.6 (1.5–1.7)		0.92 (0.86–0.99)	59,538	0.7 (0.6–0.8)		0.84 (0.76–0.92)
	1996	51,051	2.3 (2.2–2.4)		0.91 (0.86–0.97)	61,335	1.1 (1.0–1.1)		0.92 (0.85–1.00)
	1998	54,282	3.8 (3.6–4.0)		1.21 (1.15–1.26)	64,510	1.7 (1.6–1.8)		1.01 (0.95–1.09)
	2000	58,412	4.7 (4.5–4.9)	+3.1	1.02 (0.98–1.06)	67,105	2.1 (2.2–2.4)	+1.4	1.03 (0.98–1.09)

Vestfold	1994	41,883	1.4 (1.3–1.5)		0.79 (0.72–0.85)	50,432	0.7 (0.6–0.8)		0.88 (0.80–0.98)
	1996	44,493	2.0 (1.9–2.2)		0.81 (0.76–0.87)	52,436	1.0 (0.9–1.1)		0.90 (0.82–0.98)
	1998	47,153	3.7 (3.6–3.9)		1.03 (0.98–1.08)	54,840	1.8 (1.7–1.9)		1.07 (1.01–1.14)
	2000	49,676	4.6 (4.4–4.8)	+3.2	1.00 (0.96–1.04)	56,437	2.5 (2.3–2.6)	+1.8	1.11 (1.05–1.17)

Telemark	1994	32,226	1.4 (1.3–1.6)		0.83 (0.75–0.91)	39,653	0.8 (0.7–0.9)		1.01 (0.91–1.13)
	1996	33,862	2.4 (2.2–2.6)		0.96 (0.89–1.02)	40,879	1.1 (1.0–1.2)		0.99 (0.90–1.09)
	1998	35,512	4.5 (4.3–4.8)		1.24 (1.18–1.31)	42,284	2.1 (2.0–2.2)		1.23 (1.15–1.31)
	2000	37,542	5.6 (5.4–5.9)	+4.2	1.22 (1.17–1.27)	43,194	2.8 (2.6–3.0)	+2.0	1.24 (1.17–1.32)

Aust-	1994	19,428	2.3 (2.1–2.5)		1.32 (1.20–1.45)	24,177	1.3 (1.1–1.4)		1.53 (1.37–1.72)
Agder	1996	20,831	3.4 (3.2–3.7)		1.37 (1.27–1.47)	25,477	1.8 (1.7–2.0)		1.62 (1.47–1.77)
	1998	21,836	4.6 (4.3–4.9)		1.25 (1.18–1.34)	26,478	2.2 (2.0–2.4)		1.30 (1.19–1.41)
	2000	22,970	5.2 (4.9–5.5)	+2.9	1.14 (1.07–1.20)	26,985	3.0 (2.8–3.2)	+1.7	1.32 (1.23–1.42)

Vest-	1994	29,399	2.0 (1.8–2.1)		1.13 (1.04–1.22)	36,184	1.1 (1.0–1.2)		1.30 (1.17–1.44)
Agder	1996	30,912	2.6 (2.4–2.8)		1.04 (0.97–1.12)	37,735	1.7 (1.5–1.8)		1.44 (1.33–1.56)
	1998	32,761	4.2 (4.0–4.4)		1.15 (1.09–1.21)	39,339	2.5 (2.4–2.7)		1.49 (1.40–1.59)
	2000	34,905	4.9 (4.6–5.1)	+2.9	1.06 (1.01–1.11)	40,813	3.2 (3.0–3.4)	+2.1	1.43 (1.35–1.51)

*Norway was used as reference.

**Table 5:** Cumulative incidence (%) of sickness absence with different psychiatric diagnostic groups in 1994,1996,1998 and 2000 (Age-adjusted data).

NORWAY	Year	No. of sickness insured	Psychoses (95% CI)	Anxiety disorders (95% CI)	Neurotic conditions (95% CI)	Depression (95% CI)	Personality disorders (95% CI)	Alcohol and drug disorders (95% CI)
Women	1994	922,684	0.05 (0.05–0.05)	0.19 (0.19–0.19)	0.27 (0.27–0.27)	1.21 (1.19–1.23)	0.01 (0.01–0.01)	0.02 (0.02–0.02)
	1996	966,782	0.07 (0.07–0.07)	0.21 (0.21–0.21)	0.45 (0.45–0.45)	1.74 (1.72–1.77)	0.01 (0.01–0.01)	0.02 (0.02–0.02)
	1998	1,014,628	0.08 (0.08–0.08)	0.31 (0.30–0.31)	0.76 (0.76–0.76)	2.45 (2.42–2.48)	0.02 (0.02–0.02)	0.02 (0.02–0.02)
	2000	1,063,423	0.10 (0.10–0.10)	0.35 (0.34–0.35)	1.11 (1.09–1.13)	3.01 (3.00–3.04)	0.01 (0.01–0.02)	0.02 (0.02–0.03)
Increase			+0.05	+0.16	+0.84	+1.8	-----	0
Men	1994	1,089,991	0.05 (0.04–0.05)	0.10 (0.10–0.10)	0.13 (0.13–0.13)	0.47 (0.47–0.47)	0.01 (0.01–0.01)	0.06 (0.06–0.06)
	1996	1,134,437	0.05 (0.05–0.05)	0.11 (0.11–0.11)	0.21 (0.21–0.21)	0.69 (0.69–0.69)	0.01 (0.01–0.01)	0.07 (0.07–0.07)
	1998	1,181,174	0.07 (0.07–0.07)	0.17 (0.17–0.17)	0.35 (0.35–0.35)	1.01 (1.01–1.03)	0.01 (0.01–0.01)	0.08 (0.08–0.08)
	2000	1,219,338	0.09 (0.09–0.09)	0.20 (0.19–0.20)	0.54 (0.54–0.54)	1.31 (1.29–1.33)	0.01 (0.01–0.01)	0.09 (0.09–0.09)
Increase			+0.04	+0.10	+0.41	+0.84	0	+0.03
